# Volume XXX.—Alpha

**Published:** 1890-08

**Authors:** 


BUFFALO MEDICAL AND SURGICAL JOURNAL

A MONTHLY REVIEW OF MEDICINE AND SURGERY.

*EDITORS:*

THOMAS LOTHROP, M. D. - - WM. WARREN POTTER, M. D.

All communications, whether of a literary or business character, should be addressed to the editors : 284 Franklin Street, Buffalo, N. Y.

Vol. XXX. AUGUST, 1890. No. 1.

### Editorial.

### VOLUME XXX.—ALPHA.

The new volume of this Journal begins with this (August) number, and we present it in an entirely new dress. The type with which it is printed was cast especially for this magazine, and will be used only in printing it. This will insure to our readers clear letterpress for many years. The paper we now print it on is of a quality to add to the elegance of our pages.

To our heretofore efficient editorial staff two names are added that will, we feel sure, result in further extending the usefulness as well as increasing the quality of the Journal.

Professor John A. Miller was an occasional contributor to the last volume, and his graceful pen will be found in frequent use in this, with even greater force and interest.

Dr. DeLancey Rochester is already well known to the profession of Buffalo and vicinity, and his medical writings have been such as to command attention. He will be heard from in the future in a way, we predict, that will please and interest our readers.

To Drs. James Wright Putnam, John Parmenter and Frank Hamilton Potter, we are indebted for valuable aid during the past year, and they will continue in their several fields of usefulness, with even increasing energy and ability.

We shall continue our efforts to make the Journal a pride to the City which nurtures and shelters it, and a welcome visitor to our patrons and readers wherever found — to which end we invoke the good will and support of the medical profession, in whose interest alone it is published.

